# Arthropod communities on hybrid and parental cottonwoods are phylogenetically structured by tree type: Implications for conservation of biodiversity in plant hybrid zones

**DOI:** 10.1002/ece3.3146

**Published:** 2017-06-22

**Authors:** Karl J. Jarvis, Gerard J. Allan, Ashley J. Craig, Rebecca K. Beresic‐Perrins, Gina Wimp, Catherine A. Gehring, Thomas G. Whitham

**Affiliations:** ^1^ School of Forestry Northern Arizona University Flagstaff AZ USA; ^2^ Biology Department Southern Utah University Cedar City UT USA; ^3^ Department of Biological Sciences Northern Arizona University Flagstaff AZ USA; ^4^ Merriam‐Powell Center for Environmental Research Flagstaff AZ USA; ^5^ Department of Biology Georgetown University Washington DC USA

**Keywords:** arthropod phylogenetics, common garden, community genetics, community phylogenetics, foundation species, hybridization

## Abstract

Although hybridization in plants has been recognized as an important pathway in plant speciation, it may also affect the ecology and evolution of associated communities. Cottonwood species (*Populus angustifolia* and *P. fremontii*) and their naturally occurring hybrids are known to support different plant, animal, and microbial communities, but no studies have examined community structure within the context of phylogenetic history. Using a community composed of 199 arthropod species, we tested for differences in arthropod phylogenetic patterns within and among hybrid and parental tree types in a common garden. Three major patterns emerged. (1) Phylogenetic diversity (PD) was significantly different between arthropod communities on hybrids and Fremont cottonwood when pooled by tree type. (2) Mean phylogenetic distance (MPD) and net relatedness index (NRI) indicated that communities on hybrid trees were significantly more phylogenetically overdispersed than communities on either parental tree type. (3) Community distance (*D*_pw_) indicated that communities on hybrids were significantly different than parental species. Our results show that arthropod communities on parental and hybrid cottonwoods exhibit significantly different patterns of phylogenetic structure. This suggests that arthropod community assembly is driven, in part, by plant–arthropod interactions at the level of cottonwood tree type. We discuss potential hypotheses to explain the effect of plant genetic dissimilarity on arthropod phylogenetic community structure, including the role of competition and environmental filtering. Our findings suggest that cottonwood species and their hybrids function as evolutionarily significant units (ESUs) that affect the assembly and composition of associated arthropod communities and deserve high priority for conservation.

## 1 INTRODUCTION

Plant hybridization is believed to have played an important role in the diversification and speciation of many plant species (Hegarty & Hiscock, 2005; Mallet, 2007; Rieseberg, 1997). Hybridization also has been shown to have important ecological consequences for communities of dependent organisms (Whitham et al., 1999) and their interactions (Busby et al., 2015). Recent studies in hybridizing cottonwoods, willows, and oaks, for example, have shown that hybridization can influence both community composition (Bangert, Allan, et al., 2006; Wimp et al., 2004) and the evolutionary trajectory and speciation of dependent community members (Evans, Allan, Shuster, Woolbright, & Whitham, 2008). However, we still do not understand how these dependent communities are assembled, which is essential to determining drivers of community assembly, composition, and the structure of interacting networks of related species (Barbour et al., 2016; Lamit et al., 2015; Lau, Keith, Borrett, Shuster, & Whitham, 2016). One possibility is that assembly, interaction networks, and composition are a function of the evolutionary relationships among community members and that phylogenetic history influences shared community space due to shared characteristics among community members. Hence, a phylogenetic approach to understanding community assembly and structure may help to differentiate among alternative hypotheses of communities as random constructs (Hubbell, 2001) or whether evolved relationships better explain observed patterns of community composition and structure (Webb, Ackerly, McPeek, & Donoghue, 2002).

Applying phylogenetic metrics to community ecology provides a means for testing hypotheses of processes that drive community assembly (Webb et al., 2002). One such hypothesis, environmental filtering, suggests that communities are assembled because of species’ ability to occupy a particular environment, due to ecologically similar character traits arising from shared common ancestry (Emerson & Gillespie, 2008); this results in communities of closely related species, which can be considered *phylogenetically clustered* with corresponding low phylodiversity (Vamosi, Heard, Vamosi, & Webb, 2009). An alternative hypothesis, interspecific competition, suggests that species interactions largely determine which species occupy shared niche space, resulting in a community of more distantly related or *phylogenetically overdispersed* species with high phylodiversity. Both hypotheses have been invoked to explain phylogenetic patterns in many plant, animal, and microbial communities (Narwani, Matthews, Fox, & Venail, 2015). Although specific mechanisms driving phylogenetic structure are often unclear, documenting such patterns is a step toward understanding the degree to which evolutionary history contributes to community assembly, structure, and composition (Gerhold, Cahill, Winter, Bartish, & Prinzing, 2015).

We examined community phylogenetic structure in arthropods, which, despite their diversity (Brusca, Moore, & Shuster, 2016), have been little studied from a community phylogenetic perspective, and never in regard to parental or hybrid host plants. One study (Weiblen, Webb, Novotny, Basset, & Miller, 2006) examined host preference by arthropods, but focused on phylogenetic relationships of host plants rather than the arthropods themselves. Dinnage, Cadotte, Haddad, Crutsinger, and Tilman (2012) examined the relationship between phylogenetic diversity of arthropod communities on different plant species and showed a strong link between host plant diversity and arthropod phylogenetic diversity. Lessard, Fordyce, Gotelli, and Sanders (2009) examined community phylogenetic structure in ants, but not in relation to associated host plants. Lind, Vincent, Weiblen, Cavender‐Bares, and Borer (2015) studied phylogenetic patterns of predator and herbivore community members in a grassland ecosystem. To our knowledge, our study is the first to examine phylogenetic patterns of arthropod communities on hybrid and parental trees and the implications these patterns have for understanding the evolutionary significance of community assembly and structure in foundation trees that are drivers of biodiversity and associated ecosystem processes (Whitham et al., 2006, 2008).

We used a plant hybrid system consisting of two cottonwood species (*Populus fremontii* and *P. angustifolia*) and their naturally occurring F_1_ hybrids (Box 1). Observational and experimental evidence (Bangert, Allan, et al. 2006; Bangert, Turek, et al. 2006; Whitham et al., 1999; Wimp, Martinsen, Floate, Bangert, & Whitham, 2005; Wimp et al., 2004, 2007) shows that hybridization in cottonwoods drives arthropod community diversity and composition. Given that hybrids combine the genomes of two divergent species and tend to host the herbivores of their parents in equal or greater abundances (Dungey, Potts, Whitham, & Li, 2000; Strauss, 1994; Whitham et al., 1999), we hypothesized that hybrids would show greater phylogenetic diversity (Faith, 1996) and a more phylogenetically overdispersed pattern than either parent species, which may provide a more uniform environment than do hybrid trees. Alternatively, abundance and species diversity in hybrids may not result in greater phylogenetic diversity or overdispersion, because hybrids may not provide an environment that selects for unique community phylogenetic structure. Nevertheless, we considered this parental–hybrid system an important test case for examining community phylogenetic patterns as they relate to arthropod colonization of cottonwoods, because hybrid trees are known to differ from their parental species in diverse functional traits ranging from phytochemistry (Rehill et al., 2006), phenology (Floate, Kearsley, & Whitham, 1993), architecture (Bailey et al., 2004), productivity (Lojewski et al., 2009), and soil carbon fluxes (Lojewski et al., 2012). Importantly, when phytochemicals, plant ontogeny, induction, and seasonal gradients are combined into a multivariate functional trait analysis, hybrids and their parental species have been found to be different from one another (Holeski, Hillstrom, Whitham, & Lindroth, 2012). These differences affect diverse communities of organisms from microbes to vertebrates (e.g., review by Whitham et al., 1999). We are aware of no studies that have examined how hybridization affects phylogenetic relationships of any one community and ours is the first to examine how hybridization impacts community phylogenetic structure in arthropods.

Box 1 **The cottonwood‐arthropod system**

1 Cottonwoods are well known for their ability to attract and support a rich flora and fauna associated with riparian ecosystems (Whitham et al., 1999). Often referred to as *foundation species* (Bangert et al., 2008; Dayton, 1972; Ellison et al., 2005), cottonwood genotypes within and among species are linked to numerous dependent communities, including trophic structure in insects and birds (Bailey, Wooley, Lindroth, & Whitham, 2006), diversity in understory plant communities (Adams, Goldberry, Whitham, Zinkgraf, & Dirzo, 2011; Lamit et al., 2011), and networks of interacting communities ranging from canopy arthropods, leaf pathogens, lichens, ectomycorrhizae, and decomposing soil bacteria and fungi (Wimp et al., 2005, 2007).

Cottonwoods frequently hybridize where two or more species overlap, so extensive hybrid zones are common (Eckenwalder, 1984). Increased genetic variation in these hybrid zones influence biodiversity in soil microbial communities (Schweitzer et al., 2011) and arthropod communities at both local (Bangert, Allan, et al., 2006; Wimp et al., 2004) and regional scales (Bangert et al., 2008). Hybridization has also been linked to the evolution of cryptic speciation in bud‐galling mites (Evans et al., 2008) and a fungal pathogen (Newcombe, Stirling, McDonald, & Bradshaw, 2000). An important consequence of this dominant influence of cottonwoods on ecosystems is their impact on ecosystem processes such as nutrient cycling (Fischer, Hart, Schweitzer, Selmants, & Whitham, 2010; Leroy & Marks, 2006; Schweitzer et al., 2011).

## 2 MATERIALS AND METHODS

### 2.1 Sampling

We studied arthropod communities from a natural hybrid zone along the Weber River, UT, where *P*. *fremontii* (Fremont cottonwood) and *P*. *angustifolia* (narrowleaf cottonwood) naturally hybridize. For the community component of our study, we used data originally published by Wimp et al. (2005, 2007) collected from 2001 to 2003 on naturally colonized trees in a common garden that had been established for 9 years; we combined those data with unpublished data that we collected in 2000 using the same methods. Using a common garden is important because it standardizes the environment so that any observed differences among individual genotypes and their replicated clones are due to their genetic differences rather than environmental differences (Wimp et al., 2005). Sampled trees consisted of three tree types: *P. fremontii*,* P*. *angustifolia*, and F_1_ hybrids between *P*. *fremontii* and *P*. *angustifolia*. We also included backcross hybrids that result from crosses between F_1_ hybrids and *P. angustifolia* [backcrossing in this system is generally unidirectional toward *P*. *angustifolia* (Keim, Paige, Whitham, & Lark, 1989; Martinsen, Whitham, Turek, & Keim, 2001; but see also Hersch‐Green, Allan, & Whitham, 2014)]. However, molecular genetic data (Zinkgraf, 2012) show that backcrossed trees are indistinguishable from *P. angustifolia*, so we pooled data from backcrossed trees and *P. angustifolia*. The identity of individual hybrid trees was previously confirmed using RFLP data (Martinsen et al., 2001).

### 2.2 Composite phylogenetic reconstruction

**Figure 1:**
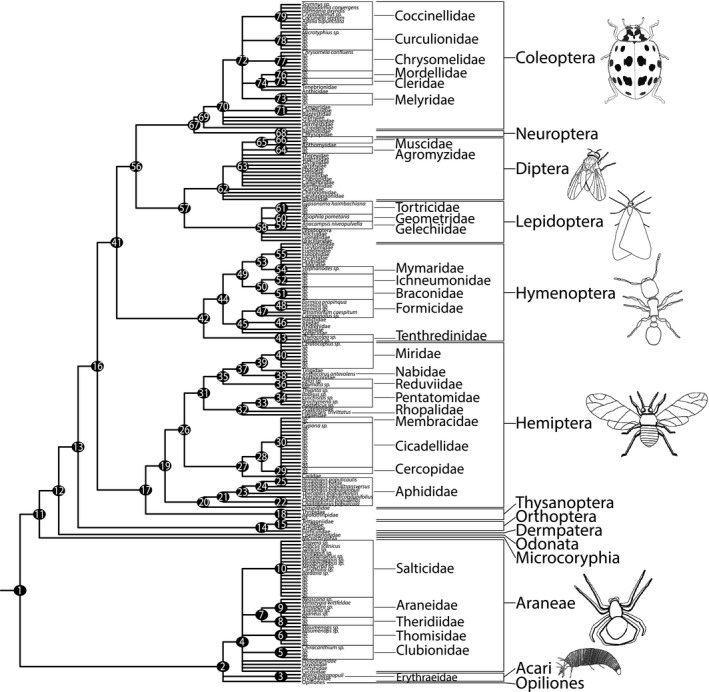
Here, we show a composite phylogeny summarizing relationships found by Misof et al. (2014) and additional well‐supported analyses. Each phylogenetic relationship is accompanied by a numbered node that corresponds to sources summarized in Appendix S1

Generating a comprehensive de novo phylogeny for community phylogenetic studies is a challenging task, especially if the community is large and molecular data are either scarce or incomplete for all operational taxonomic units (OTUs). In such cases, literature‐based supertree phylogenies can provide an accurate assessment of community phylogenetic structure, especially if the resultant phylogeny is based on multiple datasets and congruent topologies at high taxonomic rank (e.g., family and above; Beaulieu, Ree, Cavender‐Bares, Weiblen, & Donoghue, 2012). To this end, we generated a composite phylogeny based on a recent phylogenomic analysis consisting of over 1,400 protein‐coding genes (Misof et al., 2014) and arthropod phylogenies including both molecular and morphological data that represent the most comprehensive and robust phylogenies of arthropod lineages to date (Figure 1).

In addition to Misof et al. (2014), we included 39 additional studies (Appendix S1) to increase phylogenetic resolution for arachnids and intra‐ordinal resolution for insects. Using Mesquite v. 2.75 (Maddison & Maddison, 2010), we constructed a topology that summarizes relationships among the 199 individuals from the communities we studied. We included studies that used individual gene surveys, ≥100 morphological characters, or a combination of molecular and morphological data. We assumed that taxa were monophyletic if they were supported by studies that met the following criteria: (1) formal phylogenetic analyses had been performed on the data; (2) at least three species were present from the same taxon (generally congeneric or confamilial species), (3) analyses were supported by more data than any alternative analyses. We collapsed nodes for species within taxa that were not supported under these criteria. We also assumed that taxa in the composite phylogeny were monophyletic with respect to species from the same clades in our dataset; this was partly due to the few members of our dataset that were identified to species, and partly due to the lack of representation of species from our dataset that were also present in existing phylogenetic studies. Our composite phylogeny represents currently accepted interpretations of phylogenetic relationships for major extant insect orders and other arthropods included in our arthropod community. Our study primarily concerns ordinal relationships, as deep‐level relationships are most important in community phylogenetic analyses (Swenson, 2009), an approach that is also consistent with previous studies focused at higher taxonomic levels (Lessard et al., 2009).

We performed analyses on multiple branch length scenarios, because branch length estimates from multiple data sources would be incompatible. We set branch lengths in two ways in Mesquite: (1) equal branch lengths between all neighboring nodes, and (2) equal branch lengths between neighboring nodes except that we lengthened branches to the most basal nodes to make the phylogeny ultrametric. We randomized the branch lengths ten additional times to provide a means to test the sensitivity of the community phylogenetic analyses to branch length differences. We did this by adding values randomly selected from a uniform distribution between −0.99 and 0.99 to the two types of branch lengths using R (R Development Core Team and R Core Team 2016) package *ape* (Paradis, Claude, & Strimmer, 2004). We focused our results on the ultrametric model because it assumes a constant rate of evolution, a common assumption and potentially a more appropriate null model of evolution than the alternative (Gaunt & Miles, 2002).

### 2.3 Intra‐ and intercommunity metrics

We conducted community phylogenetic analyses on communities from individual tree types as well as communities pooled by tree type and year. We controlled for key factors: number of species per community, abundance of each species, and topology of the arthropod phylogeny. We used R package *picante* v. 1.4 (Kembel et al., 2010) to calculate phylogenetic diversity (Faith, 2006) and intra‐ and intercommunity phylogenetic structure (Box 2). For intracommunity phylogenetic structure, we calculated abundance‐weighted mean pairwise distance (MPD; Webb et al., 2002) to account for the effect of numbers of individuals in each taxon on phylogenetic diversity. We also calculated net relatedness index (NRI; Webb et al., 2002) from MPD by standardizing effect sizes by the standard error of 999 null models that maintained species richness and changing the sign of the results.

Box 2 1 *Phylogenetic Diversity* (PD) is the sum of distances of branch lengths between all pairs of individuals in a community (Faith, 2006). This metric is meant to be an approximate metric that includes species richness and is not standardized.

*Mean Phylogenetic Distance* (MPD) is the mean distance between each pair of individuals in a community (Webb et al., 2002). This metric is not standardized but corrects for species richness.

*Net Relatedness Index* (NRI) is a standardized version MPD, with the sign reversed (Webb et al., 2002). The formula for NRI includes the MPD of multiple randomized communities, which can allow it to account for abundance of each species. Positive values indicate phylogenetic clustering, and negative values indicate phylogenetic overdispersion.

*Community Distance* (*D*_pw_), or mean pairwise distance between communities, is similar to MPD, but compares mean phylogenetic distance between each pair of individuals from different communities. The output of this procedure is a distance matrix, which can be analyzed using ordinations or other methods.

We used linear mixed‐effect (LME) modeling to compare effects of tree type on PD, MPD, and NRI at the levels of individual trees and pooled trees using R package *nlme* (Pinheiro et al. 2015). All LME models included tree type as a fixed effect. Models of metrics at the individual (nonpooled) tree level included branch length, year, and tree as random effects, and models of metrics at the pooled tree level included branch length and year as random effects. We estimated parameters using restricted maximum likelihood. We calculated 95% confidence intervals (CI) to infer differences among tree types in each model.

To assess intercommunity relationships, we calculated mean pairwise distance among communities (*D*_pw_; Feng et al., 2012; Webb, Ackerly, & Kembel, 2008). To visualize the relationships in distance matrices from *D*_pw_ analyses, we performed ordinations via nonmetric multidimensional scaling on both individual and pooled communities in two dimensions using R package *MASS* (Ripley et al., 2013). We constructed 95% confidence ellipses around results with the same tree type. To test for differences by tree type and year, we performed permutational multivariate analyses of variance (PERMANOVA) using the *adonis* function in R package *vegan* (Oksanen et al. 2015). We tested tree type and year as predictors for community distance among communities on individual trees and communities pooled by tree type and year.

In MPD, MRI, and *D*_pw_ analyses, we weighted results by abundance within each species to account for greater ecological impact of more abundant species. For *D*_pw_, we also weighted all species equally for comparison to abundance‐weighted analyses. We considered statistical significance to be at α = .05.

## 3 RESULTS

The phylogeny consisted of 199 terminals comprising 11 orders of insects and three orders of arachnids, based on Misof et al. (2014) and 39 additional studies (Appendix S1). These studies support the monophyly of Insecta and major clades within it, particularly Polyneoptera (Orthoptera, Dermaptera), Condylognatha (Hemiptera, Thysanoptera), Holometabola (Coleoptera, Hymenoptera, Diptera, Lepidoptera, Neuroptera), and the individual insect orders. Misof et al. (2014) also provided strong support for relationships within (e.g., basal Hymenoptera within Holometabola) and among these clades (e.g., Condylognatha + Holometabola). Overall, 79 of 198 possible phylogenetic relationships were resolved.

The total number of individuals in all species in the analysis was 19,022, and the number of individuals in all species per year was 3,206, 3,149, 7,795, and 4,872 for 2000–2003. A mean of 31.9% of all species were present within pooled communities (all arthropods on trees of the same type within a year); of those species, mean abundance was 27.1 and varied from 2.0 to 42.5. For nonpooled communities (all arthropods on an individual tree within a year), a mean of 7.5% of species were present; of those species, mean abundance per species was 2.6 and varied from 1.1 to 15.5.

**Figure 2:**
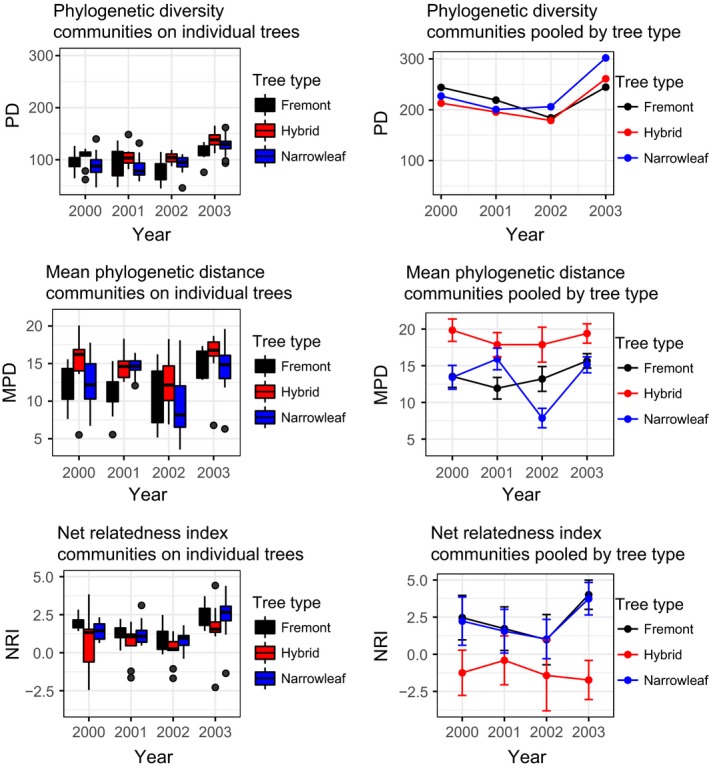
Phylogenetic diversity (PD), mean phylogenetic distance (MPD), and net relatedness index (NRI) for individual trees and for communities pooled by tree type across all years. Greater PD and MPD values indicate greater mean phylogenetic distance among members of a community. NRI values >0 indicate phylogenetic clustering, and values <0 indicate phylogenetic overdispersion. Error bars for MPD and NRI are standard deviations of results of 999 null models generated in standardizing effect size for NRI

We evaluated phylogenetic diversity (PD) in two ways: using pooled and nonpooled datasets (Figure 2). When communities were not pooled, LME models of PD indicated that confidence intervals (CI) contained zero for both Fremont (−3.37, 1.90) and narrowleaf (−2.13, 2.41) cottonwoods, suggesting no significant differences among communities on individual trees (Table 2). When communities were pooled by tree type, models of PD contained zero for narrowleaf (−2.62, 13.12) but not Fremont cottonwoods (0.19, 15.79), suggesting that when tree types are treated as communities, there may be greater differences in PD between hybrids and Fremont cottonwoods. We found that CI for species richness did not contain zero for analyses of either individual (18.48, 21.15) or pooled communities (1.36, 1.72).

Mean phylogenetic distance analyses resulted in more overdispersion for arthropod communities on hybrid trees than communities on Fremont or narrowleaf, which was especially apparent when we pooled communities by tree type (Figure 2). Communities on individual hybrid trees had significantly higher MPD than Fremont trees (−3.33, −0.34), but did not differ significantly from narrowleaf trees (−2.22, 0.37) (Table 2). Communities pooled by tree type indicated significantly greater MPD values for hybrids than both Fremont (−5.18, −1.48) and narrowleaf trees (−5.23, −1.53).

Net relatedness index results indicated that communities from individual hybrid trees tended not to be significantly different from neutral expectations, but that some communities from Fremont and narrowleaf trees were significantly phylogenetically clustered (Table 1, Figure 2). NRI values were nonsignificant (*p* > .05) in 142 of 160 communities when pooled by tree type and year. Communities from hybrid trees had the highest mean *p*‐values, indicating they are not significantly different than neutral expectations; however, Fisher's method for combining *p*‐values indicated that all tree type–year combinations were significantly different than neutral expectations, except for communities on hybrids from 2003. LME models indicated that the 95% CI of NRI of communities from individual hybrid trees was not significantly lower than communities from Fremont trees (1.03, 0.00) or narrowleaf trees (0.48, −0.41; Table 2). However, when pooled by tree type, arthropod communities on hybrid trees were significantly different than communities from Fremont (1.94, 3.73) and narrowleaf trees (1.31, 3.11).

**Table 1:** Our analyses generally indicated greater mean phylogenetic distance (MPD) for communities on hybrid trees than parental trees. This was the case for both types of analyses – arthropods from individual trees analyzed separately and all arthropods from the same tree type pooled by year for analysis. Net relatedness index (NRI, standardized effect size of MPD) indicated that analyses of communities from individual trees did not differ from null expectations, except for the means of Fremont communities in 2 years. However, NRI indicated significant clustering (α = .05) for communities pooled by tree type and year for Fremont (3 years) and narrowleaf (2 years). NRI of communities from hybrid trees did not differ significantly from random, compared to null expectations, with much higher *p*‐values than communities from Fremont or narrowleaf trees

Year	Type	Individual Means								Pooled						
		Species richness	Phylogenetic diversity	Mean phylogenetic distance	Random means	Random *SD*	Net relatedness index	Mean *p*‐value	Fisher's *p*‐value	Species richness	Phylogenetic diversity	Mean phylogenetic distance	Random means	Random *SD*	Net relatedness index	*p*‐value
2000	Fremont	16.5	96	12.4	15.8	1.8	1.9	0.044	0.00	66	244	13.5	17.2	1.5	2.5	0.01
	Hybrid	19.7	105	15.3	16.6	1.4	0.8	0.332	0.00	62	213	19.8	17.9	1.5	−1.2	0.90
	Narrowleaf	16.1	89	12.7	15.0	1.6	1.4	0.112	0.00	66	227	13.4	17.0	1.6	2.2	0.02
2001	Fremont	15.9	93	11.0	13.0	1.5	1.3	0.145	0.00	56	219	11.9	14.5	1.5	1.7	0.03
	Hybrid	19.2	106	14.6	15.6	1.8	0.6	0.309	0.02	56	196	17.9	17.2	1.6	−0.4	0.61
	Narrowleaf	13.6	84	14.6	16.7	1.9	1.2	0.165	0.00	52	201	15.9	18.2	1.5	1.6	0.07
2002	Fremont	12.0	79	10.5	12.3	1.6	1.0	0.234	0.01	40	184	13.2	14.9	1.7	1.0	0.16
	Hybrid	17.4	103	12.4	12.7	1.6	0.2	0.421	0.36	46	179	17.9	14.5	2.4	−1.4	0.97
	Narrowleaf	14.2	93	9.3	10.6	1.6	0.8	0.232	0.01	49	206	7.9	9.2	1.3	1.0	0.14
2003	Fremont	23.3	114	14.9	18.3	1.4	2.4	0.032	0.00	75	245	15.7	19.6	1.0	4.0	0.00
	Hybrid	29.5	138	16.0	17.8	1.1	1.6	0.161	0.00	85	261	19.4	17.1	1.3	−1.7	0.95
	Narrowleaf	27.0	128	14.5	17.8	1.4	2.5	0.066	0.00	108	302	15.1	19.2	1.1	3.7	0.00

**Table 2:** Linear mixed‐effect (LME) models of the effect of cottonwood tree type on phylogenetic metrics of arthropod communities indicate significant differences of among tree types. We built LME models based on three metrics of communities on individual trees and communities pooled by tree type. We present parameter estimates, standard error (*SE*), test statistic (*t* value), and 95% confidence intervals (CI). Because the variables were calculated as contrasts, the first variable estimate is based on the mean value due to hybrid tree type, and Fremont, narrowleaf, and species richness estimates indicate the expected difference due to those variables

Analysis	Metric	Variable	Estimate	SE	*t* value	CI
Communities on individual trees	PD	Intercept (Hybrid)	26.43	22.55	3.51	(24.73, 133.55)
		Fremont	−0.73	1.34	−0.55	(−3.37, 1.90)
		Narrowleaf	0.15	1.15	0.13	(−2.13, 2.41)
		Species Richness	**2.86**	**0.64**	**31.05**	**(18.48, 21.15)**
	MPD	Intercept (Hybrid)	11.06	3.02	3.66	(3.79, 18.32)
		Fremont	−**1.83**	**0.76**	−**2.40**	**(**−**3.33,** −**0.34)**
		Narrowleaf	−0.92	0.66	−1.39	(−2.22, 0.37)
	NRI	Intercept (Hybrid)	1.28	0.35	3.61	(2.00, 0.55)
		Fremont	0.51	0.26	1.96	(1.03, 0.00)
		Narrowleaf	0.04	0.23	0.17	(0.48, −0.41)
Communities pooled by tree type	PD	Intercept (Hybrid)	73.40	47.99	1.53	(−41.64, 188.43)
		Fremont	**7.99**	**4.10**	**1.95**	**(0.19, 15.79)**
		Narrowleaf	5.25	4.13	1.27	(−2.62, 13.12)
		Species Richness	**1.54**	**0.09**	**16.22**	**(1.36, 1.72)**
	MPD	Intercept (Hybrid)	13.84	3.61	3.83	(5.16, 22.52)
		Fremont	−**3.33**	**0.95**	−**3.52**	**(**−**5.18,** −**1.48)**
		Narrowleaf	−**3.38**	**0.95**	−**3.57**	**(**−**5.23,** −**1.53)**
	NRI	Intercept (Hybrid)	−0.30	0.51	−0.58	(−1.38, 0.78)
		Fremont	**2.83**	**0.46**	**6.20**	**(1.94, 3.73)**
		Narrowleaf	**2.21**	**0.46**	**4.84**	**(1.31, 3.11)**

Variables with CI estimates that include zero are bolded.

Net relatedness index of communities pooled by tree type and year indicated that parental trees were significantly clustered in five of eight cases evaluated by year. Communities on Fremont indicated significantly positive NRI in three of 4 years; communities on narrowleaf were significantly positive in 2 of 4 years, with a third year (2001) marginally higher than the α level (*p* = .067). Communities on hybrids pooled by year had negative NRI values and much higher *p*‐values than parental trees (mean *p* = .86), indicating lack of differences from neutral expectations.

**Figure 3:**
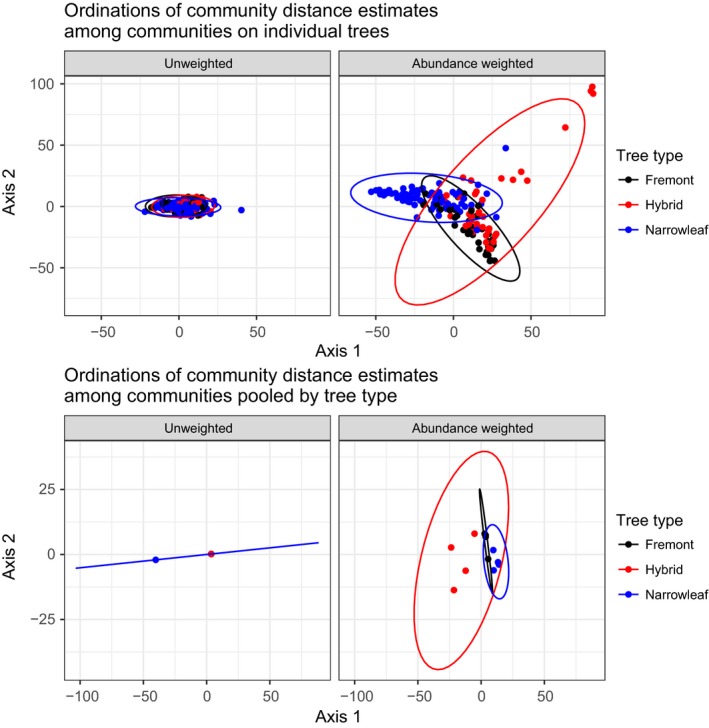
Nonmetric multidimensional scaling of community distance results indicate high overlap among 95% confidence ellipses when community phylogenetic distances are calculated without weighting data by abundance. Abundance‐weighted results show greater dispersion of points and less overlap of ellipses than unweighted community distance results

Our analyses of phylobetadiversity as measured by community distance (*D*_pw_) indicate clear differences between hybrid and parental trees, but primarily when we accounted for differences in species abundance (Figure 3). When results were not weighted by abundance, confidence ellipses overlapped greatly for each of the three tree types, but when weighted by abundance, confidence ellipses overlapped much less. This result was consistent when measured for communities on individual trees and in communities pooled by tree type. We also found that communities on Fremont and narrowleaf trees were more similar to one another than were communities on hybrid trees. Moreover, variation among communities on hybrids was wider from year to year than variation among communities on either parental tree type, which is consistent with the hypothesis that parental trees provide a more uniform environment relative to hybrid trees.

PERMANOVAs supported our results from confidence ellipses around NMDS points (Table 3). Tree type had *r* ^2^ = .30 (*p* < .01) for individual trees and *r* ^2^ = .41 for communities pooled by tree type (*p* < .01) when we analyzed abundance‐weighted community distance. However, for unweighted community distance, tree type was a poorer predictor, with *r* ^2^ = .02 (*p* = .03) for individual trees, and *r* ^2^ = .18 (*p* = .78) for communities pooled by tree type. Although some differences in tree type in PERMANOVA could be due to different multivariate spread of these factors, it is clear that the results differ between abundance‐weighted and unweighted community distance.

**Table 3:** PERMANOVA results of community distance (*D*_pw_) analyses indicate the ability of variables to explain variation relative to tree type and year. We present results run on analyses of communities from individual trees and pooled by tree type and year. We also present weighting scheme (weighting), the names of the factors in the PERMANOVA (Variable), degrees of freedom (*df*), and correlation coefficient (*r* ^2^) with its *p*‐value. All analyses were from the phylogeny with ultrametric branch lengths

Community definition	Weighting	Variable	*df*	*r* ^2^	*p*‐value
Individual trees	Abundance‐weighted	Tree type	2	.3	.001
		Year	3	.07	.001
	Unweighted	Tree type	2	.02	.017
		Year	3	.04	.002
Pooled by tree type and year	Abundance‐weighted	Tree type	2	.41	.001
		Year	3	.22	.198
	Unweighted	Tree type	2	.18	.778
		Year	3	.27	.133

## 4 DISCUSSION

Our analyses show that arthropod communities, when pooled by parental and hybrid tree type, differ with respect to mean phylogenetic distance (MPD), net relatedness index (NRI), and community distance (*D*_pw_), as well as phylogenetic diversity (PD) between Fremont trees and hybrids. Importantly, we found these patterns to be generally consistent across 4 years of arthropod community surveys, suggesting that community phylogenetic structure is a stable feature among parental and hybrid trees. Below we discuss the importance of each community phylogenetic metric for understanding arthropod community assembly and structure on different cottonwood tree types.

### 4.1 Intracommunity phylogenetic metrics

Abundance‐weighted measures of MPD and NRI indicated more phylogenetic overdispersion for communities on hybrids than parental trees when we pooled communities by tree type. This finding is consistent with Wimp et al. (2004) who found that genetic variation in cottonwoods is significantly correlated with biodiversity in arthropod communities. Importantly, our study expands upon this result by demonstrating that arthropod biodiversity among trees has a phylogenetic component—one that is not apparent from correlative or clustering techniques (e.g., NMDS) that do not account for patterns arising from evolutionary relatedness among diverse taxa.

Consistent differences in community phylogenetic metrics across years suggest communities are stable, which has been documented using abundance‐weighted and unweighted analyses in plant communities (Cadotte, Dinnage, & Tilman, 2012). Community stability has been documented for narrowleaf cottonwood in which stability across years was genotype dependent; that is, some genotypes were stable in their arthropod communities across years, whereas others were not, and stability was shown to be a heritable trait (Keith, Bailey, & Whitham, 2010). Differences in phylogenetic metrics among tree types in our study suggest that communities may also be stable on Fremont and hybrid trees.

Greater differences in PD, MPD, and NRI between hybrids and parental trees when we pooled communities by tree type suggest that, as a whole, there is a greater diversity of habitat for arthropods living on hybrid trees. On the other hand, intracommunity metrics suggest that communities from individual hybrids tend to have no greater diversity than communities from individual parental trees. A possible explanation for this phenomenon is greater phenotypic diversity among hybrid genotypes, which we expected based on their more diverse parentage and genetic distance among individuals than either parental species (Whitham et al., 1999) Individual hybrid trees may not provide much, if any additional phenotypic diversity relative to individual parental trees, but diversity among hybrid trees as a whole may be greater.

Our finding that arthropod communities on hybrid trees were overdispersed relative to parental trees suggests that hybrid and parental trees offer contrasting environments and likely play unique roles in determining the assembly and corresponding phylogenetic structure of their respective arthropod communities. At this stage, however, it is unclear what mechanisms drive these patterns. Traditionally, phylogenetic overdispersion has been attributed to competitive interactions among species within a community (Webb et al., 2002), a mechanism first described by Darwin (1859) regarding niche space that is shared among different species.

On the other hand, phylogenetically clustered communities (which we observed on parental tree types) may arise because members of the community share traits that are critical to their survival and overall fitness, a pattern most often attributed to habitat or environmental filtering (Goberna, Navarro‐Cano, Valiente‐Banuet, García, & Verdú, 2014; Verdú & Pausas, 2007). In communities on parental trees, one possibility for the observed pattern of phylogenetic clustering is that parental species provide a more uniform, genetically based phytochemical environment than hybrids, filtering the arthropod community for specific physiological requirements shared by closely related arthropod species (Wiens & Graham, 2005). This process could arise from differences in chemical defense traits, for which each parent species is divergent; narrowleaf cottonwood is typically high in tannins, while Fremont cottonwood is low in tannins, but high in salicortin content (Rehill et al., 2006). Morphological traits that define each parental species could also influence environmental filtering processes (Eckenwalder, 1977).

Alternatively, competition may play a role in driving both phylogenetic clustering and overdispersion. Numerous studies in this system show that the experimental removal or addition of one species results in competitive release or decline of many other species (e.g., Busby et al., 2015; Waltz et al. 1997). Furthermore, Mayfield and Levine (2010) suggested that competition in homogeneous environments could lead to phylogenetic clustering due to unique characteristics found only in a few clustered clades that outcompete other taxa due to quicker or more efficient use of resources, which is consistent with our finding of phylogenetically clustered communities on parental trees. On the other hand, competition in heterogeneous environments could lead to phylogenetic overdispersion due to adaptations that fill a wider diversity of niches. Our results for overdispersed communities on hybrid trees fit this model, because hybrid trees likely provide more diverse habitat for arthropods than parental trees. Nevertheless, competition may not always explain phylogenetic patterns, as in the case of Alexandrou et al. (2015) who demonstrated that competition did not predict patterns of evolutionary relatedness in both natural and experimental algal communities.

Various other mechanisms could also drive community phylogenetic patterns. For example, phylogenetic distance of the study taxa could also contribute to phylogenetic clustering and overdispersion (Horn, Caruso, Verbruggen, Rillig, & Hempel, 2014), as could facilitation or antagonistic/competitive interactions (Thonar, Frossard, Šmilauer, & Jansa, 2014). Moreover, phylogenetic overdispersion may arise from convergence of distantly related species if different species have converged on similar traits supporting their coexistence within a particular habitat (e.g., Cavender‐Bares, Ackerly, Baum, & Bazzaz, 2004). Although our analyses cannot identify a specific mechanism responsible for the patterns observed on different cottonwood tree types, it is nonetheless clear that community phylogenetic structure exists and varies based on cottonwood tree type.

### 4.2 Phylobetadiversity

Estimates of phylobetadiversity (PBD) such as D_pw_ can provide insights into the degree to which communities are evolutionarily similar to one another, thereby providing a historical component to the analysis of community similarity across space and time (Graham & Fine, 2008). Although PBD has been measured for many plant and forest tree communities (e.g., Duarte, Bergamin, Marcilio‐Silva, Seger, & Marques, 2014; Fine & Kembel, 2011), and some microbial communities (Wang et al., 2013), estimates of PBD for animal communities are less common (but see Gomez, Bravo, Brumfield, Tello, & Cadena, 2010; Losos, 1992; Rabosky, Cowan, Talaba, & Lovette, 2011). Consistent with our finding that arthropods on hybrid and parental trees differ in phylogenetic structure, we also found that communities on different trees were phylogenetically dissimilar from one another.

Because PBD quantifies phylogenetic distance among communities, it may be that differences in PBD among hybrid and parental plants reflect unique environments that arthropods encounter when forming assemblages in cottonwood hybrid zones. For example, Evans et al. (2008) found that a cryptic species of mite had evolved on hybrid cottonwoods, which was absent on either parental species. Thus, our assessment of PBD identifies genetic conditions in which patterns in community structure change across a gradient of tree types.

### 4.3 Potential causes for overdispersed communities on hybrid cottonwoods

Our observation that communities on hybrids trees are phylogenetically overdispersed suggests that competitive interactions may be driving this pattern. Strong competitive interactions among closely related species have been documented in other studies involving fungal pathogens (Gilbert & Webb, 2007), protists (Violle, Nemergut, Pu, & Jiang, 2011), various vertebrates (Cooper, Rodríguez, & Purvis, 2008; Davies, Meiri, Barraclough, & Gittleman, 2007; Kozak, Larson, Bonett, & Harmon, 2005; Lovette & Hochachka, 2006), and microbial communities (Horner‐Devine & Bohannan, 2006). One study (Lessard et al., 2009) found that native ant communities are phylogenetically altered toward overdispersion when invasive ant species alter the community, which suggests competition. Bennett, Lamb, Hall, Cardinal‐McTeague, and Cahill (2013), however, found that increased competition did not lead to phylogenetic overdispersion in a native grassland community, but instead suggested that specific conditions involving trait conservatism need to be met for overdispersion to occur. Alternatively, if traits important for habitat specialization on hybrid trees are labile and closely related species specialize on these traits, then phylogenetic overdispersion can also occur in the absence of competition (e.g., Cavender‐Bares et al., 2004; Fine, Mesones, & Coley, 2004).

Competitive exclusion experiments with pure and hybrid plants could test the hypothesis that the functional traits associated with genetic characteristics of pure and hybrid plants influence interspecific competitive interactions. These tests could also demonstrate the extent to which competitive interactions on hybrids promote phylogenetic overdispersion or clustering in arthropod communities. Such experiments on the competitive interactions among arthropods could also offer inferences into plant–arthropod interactions for other foundation trees (e.g., willows and oaks). The results of these tests could promote deeper understanding of the evolutionary basis of community assembly (Gerhold et al., 2015) and the mechanisms driving community phylogenetic structure (Cavender‐Bares, Kozak, Fine, & Kembel, 2009).

### 4.4 Conservation implications

Our study suggests that parental species and their hybrid derivatives each contribute to maximizing biodiversity arising from unique evolutionary processes based on differential phylogenetic sorting of communities. It also suggests that the loss of one parental cottonwood tree type would eliminate the generation of new hybrids, which do not breed true, or lead to the loss of hybrids, which would negatively affect the biodiversity and associated species interactions unique to hybrids. Thus, the maintenance and preservation of biodiversity are dependent upon the preservation of all three tree types, which directly relates to decisions on which units of conservation to protect (Vane‐Wright, Humphries, & Williams, 1991). Our results suggest that hybrids in particular contribute to differences in community diversity (Wimp et al., 2004, 2005), because of their association with communities that are differentially phylogenetically structured relative to parental trees (i.e., they are overdispersed). It will be important to determine if other types of plant hybrid zones show similar patterns of community phylogenetic structure, especially in the case of other foundation species, which often have large impacts on dependent community assembly and structure (e.g., hybridizing oaks; Pérez‐López, González‐Rodríguez, Oyama, & Cuevas‐Reyes, 2016). Finally, we suggest that because cottonwood hybrid zones drive phylogenetic structure in arthropods, they ought to be considered targets for conservation and protected as evolutionarily significant units that promote and maintain biological diversity (Evans et al., 2008; Floate, Godbout, Lau, Isabel, & Whitham, 2016; Whitham, Morrow, & Potts, 1991).

## CONFLICT OF INTEREST

The authors have no conflicts of interest, financial, or otherwise, which could affect their objectivity with this study.

## AUTHOR CONTRIBUTIONS

All authors contributed to developing the study design, interpretation of the analyses, and crafting of the manuscript. GW collected the arthropod community data, and TGW established and maintained the common garden used in this study. KJJ developed the phylogeny, conducted analyses, and created figures and tables, and RKBP created the arthropod illustrations. GJA and KJJ wrote the bulk of the manuscript and oversaw the writing process, and AJC formatted the manuscript.

## DATA ACCESSIBILITY

The arthropod community data analyzed in this study will be published for public use on http://datadryad.org/. The R script and code for the phylogenetic tree that we developed are included as Appendices S2 and S3.
