# Supplementary material for: Arthropod communities on hybrid and parental cottonwoods are phylogenetically structured by tree type: Implications for conservation of biodiversity in plant hybrid zones
Source: Ecol Evol. 2017 Jun 22;7(15):5909–21. doi: 10.1002/ece3.3146 (PMC5551273; doi:10.1002/ece3.3146)
Supplement: Supplementary file 1 [file ECE3-7-5909-s001.docx]

**Appendix 1.** Studies used to assemble a composite phylogeny of arthropods surveyed from Wimp et al. (2004). Node identifies the node of interest from Fig 1. Level indicates the taxonomic level, if available. Taxon indicates the taxon or taxa that are hypothesized to be monophyletic. Evidence indicates the number of loci and sequence length (bp) of nuclear DNA (nDNA), mitochondrial DNA (mtDNA), proteins (Amino acids), and coded morphological characters (Morph) for each study.

| Node | Level | Taxon | Evidence | Citation |
| --- | --- | --- | --- | --- |
| 1 | Phylum | Arthropoda | 13 mtDNA loci: 15,474 bp | (Cameron et al. 2004) |
| 2 | Subphylum | Chelicerata | 2 nDNA loci: 1,350 bp, Morph: 93 | (Wheeler & Hayashi 1998) |
| 3 | Order | Acari | 2 nDNA loci: 1,350 bp, Morph: 93 | (Wheeler & Hayashi 1998) |
| 4 | Order | Araneae | 2 nDNA loci: 1,350 bp, Morph: 93 | (Wheeler & Hayashi 1998) |
| 5 | Family | Clubionidae | Morph: 157 | (Bosselaers & Jocque 2002) |
| 6 | Family | Thomisidae | 2 nDNA , 1 mtDNA loci: 1315 bp | (Benjamin et al. 2008) |
| 7 | Superfamily | Araneioidea | 4 nDNA, 2 mtDNA loci: 4371 bp | (Blackledge et al. 2009) |
| 8 | Family | Theridiidae | 4 nDNA, 2 mtDNA loci: 4371 bp | (Blackledge et al. 2009) |
| 9 | Family | Araneidae | 4 nDNA, 2 mtDNA loci: 4371 bp | (Blackledge et al. 2009) |
| 10 | Family | Salticidae | 3 mtDNA loci: 2084 bp | (Maddison & Hedin 2003) |
| 11 | Class | Insecta | Amino acids: 413459 | (Misof et al. 2014) |
| 12 | Subclass | Pterygota | Amino acids: 413459 | (Misof et al. 2014) |
| 13 | Infraclass | Neoptera | Amino acids: 413459 | (Misof et al. 2014) |
| 14 | Superorder | Polyneoptera | Amino acids: 413459 | (Misof et al. 2014) |
| 15 | Order | Orthoptera | Amino acids: 413459 | (Misof et al. 2014) |
| 16 | - | Eumetabola | Amino acids: 413459 | (Misof et al. 2014) |
| 17 | Superorder | Paraneoptera | Amino acids: 413459 | (Misof et al. 2014) |
| 18 | Order | Thysanoptera | Amino acids: 413459 | (Misof et al. 2014) |
| 19 | Order | Hemiptera | Amino acids: 413459 | (Misof et al. 2014) |
| 20 | Suborder | Sternorrhyncha | 1 nDNA locus: 580 bp | (von Dohlen & Moran 1995) |
| 21 | Family | Aphididae | 3 mtDNA loci: 2794 bp | (Von Dohlen et al. 2006) |
| 22 | Genus | Chaitophorus | 1 nDNA locus: 646 bp | (Wieczorek & Kajtoch 2011) |
| 23 | Subfamily | Pemphigini | 2 nDNA, 2 mtDNA loci: 2752 bp | (Ortiz-Rivas & Martínez-Torres 2010) |
| 24 | Genus | Pemphigus | 2 mtDNA, 2 endosymbiont DNA loci: 4302 bp | (Abbot & Withgott 2004) |
| 25 | Subgenus | Pemphigus betae + Pemphigus populicaulis | 2 mtDNA, 2 endosymbiont DNA loci: 4302 bp | (Abbot & Withgott 2004) |
| 26 | - | Auchenorrhyncha + Heteroptera | 5 nDNA, 2 mtDNA loci: 7030 bp | (Cryan & Urban 2012) |
| 27 | Suborder | Auchenorrhyncha | 5 nDNA, 2 mtDNA loci: 7030 bp | (Cryan & Urban 2012) |
| 28 | Infraorder | Cicadomorpha | 5 nDNA, 2 mtDNA loci: 7030 bp | (Cryan & Urban 2012) |
| 29 | Family | Cercopidae | 5 nDNA, 2 mtDNA loci: 7030 bp | (Cryan & Urban 2012) |
| 30 | Superfamily | Membracoidea | 5 nDNA, 2 mtDNA loci: 7030 bp | (Cryan & Urban 2012) |
| 31 | Suborder | Heteroptera | 5 nDNA, 2 mtDNA loci: 7030 bp | (Cryan & Urban 2012) |
| 32 | Infraorder | Pentatomorpha | 2 nDNA, 2 mtDNA loci: 4186 bp | (Li et al. 2012) |
| 33 | - | Pentatomidae + Scutelleridae | 2 nDNA, 2 mtDNA loci: 3870 bp | (Grazia et al. 2008) |
| 34 | Family | Pentatomidae | 2 nDNA, 2 mtDNA loci: 3870 bp | (Grazia et al. 2008) |
| 35 | Infraorder | Cimicomorpha | 2 nDNA, 1 mtDNA loci: 3275 bp | (Tian et al. 2008) |
| 36 | Family | Reduviidae | 2 nDNA, 1 mtDNA loci: 3300 bp | (Weirauch & Munro 2009) |
| 37 | - | Anthocoridae + Nabidae + Tingidae + Miridae | 2 nDNA, 2 mtDNA loci: 4141 bp, Morph: 73 | (Schuh et al. 2009) |
| 38 | - | Anthocoridae + Nabidae | 2 nDNA, 2 mtDNA loci: 4141 bp, Morph: 73 | (Schuh et al. 2009) |
| 39 | Superfamily | Mirioidea | 2 nDNA, 2 mtDNA loci: 4141 bp, Morph: 73 | (Schuh et al. 2009) |
| 40 | Family | Miridae | 2 nDNA, 1 mtDNA loci: 3275 bp | (Tian et al. 2008) |
| 41 | - | Holometabola (Endopterygota) | Amino acids:413459 | (Misof et al. 2014) |
| 42 | Order | Hymenoptera | Amino acids:413459 | (Misof et al. 2014) |
| 43 | Family | Tenthredinidae | 2 nDNA, 1 mtDNA loci: 2584 bp | (Leppänen et al. 2012) |
| 44 | Suborder | Apocrita | 3 nDNA, 1 mtDNA loci: 8576 bp, Morph:392 | (Sharkey et al. 2012) |
| 45 | Infraorder | Aculeata | 3 nDNA, 1 mtDNA loci: 8576 bp, Morph:392 | (Sharkey et al. 2012) |
| 46 | - | Anthophila | Supermatrix of 4 nDNA loci:17,269 bp | (Hedtke et al. 2013) |
| 47 | Family | Formicidae | 1 nDNA locus: 1240 bp | (Ouellette et al. 2006) |
| 48 | Genus | Formica | 1 mtDNA locus: 1441 bp | (Goropashnaya et al. 2012) |
| 49 | Superfamily | Ichneumonoidea | 3 nDNA, 1 mtDNA loci: 8576 bp, Morph:392 | (Sharkey et al. 2012) |
| 50 | - | Ichneumonoidea + Chalcidoidea | 3 nDNA, 1 mtDNA loci: 8576 bp, Morph:392 | (Sharkey et al. 2012) |
| 51 | Family | Braconidae | 4 nDNA loci: 3982 bp | (Sharanowski et al. 2011) |
| 52 | Family | Ichneumonidae | 1 nDNA locus: 1001 bp | (Quicke et al. 2009) |
| 53 | Superfamily | Chalcidoidea | 2 nDNA loci: 6021 bp | (Munro et al. 2011) |
| 54 | Family | Mymaridae | 2 nDNA loci: 6021 bp | (Munro et al. 2011) |
| 55 | - | Pteromalidae + Eurytomidae + Eupelmidae + Eulophidae + Encyrtidae + Dryinidae + Chalcidae | 2 nDNA loci: 6021 bp | (Munro et al. 2011) |
| 56 | - | Lepidoptera + Diptera + Neuroptera + Coleoptera | Amino acids: 413459 | (Misof et al. 2014) |
| 57 | - | Diptera + Lepidoptera | Amino acids: 413459 | (Misof et al. 2014) |
| 58 | Order | Lepidoptera | Amino acids: 413459 | (Misof et al. 2014) |
| 59 | Family | Gelechiidae | 7 nDNA, 1 mtDNA loci: 6059 bp | (Karsholt et al. 2013) |
| 60 | Family | Geometridae | 7 nDNA, 1 mtDNA loci: 6157 bp | (Sihvonen et al. 2011) |
| 61 | Family | Tortricidae | 5 nDNA loci: 14,826 bp | (Regier et al. 2012) |
| 62 | Order | Diptera | Amino acids: 413459 | (Misof et al. 2014) |
| 63 | Suborder | Brachycera | 1 nDNA locus: 2600 bp | (Wiegmann et al. 2003) |
| 64 | Family | Agromyzidae | 2 nDNA, 1 mtDNA loci: 2965 bp | (Scheffer et al. 2007) |
| 65 | - | Calyptratae | 4 nDNA, 4 mtDNA loci: 7202 bp | (Kutty et al. 2008) |
| 66 | Family | Muscidae | 2 nDNA, 2 mtDNA loci: 2989 bp | (Schnell e Schuehli et al. 2007) |
| 67 | - | Coleoptera + Neuroptera | Amino acids: 413459 | (Misof et al. 2014) |
| 68 | Superorder | Neuropterida | Amino acids: 413459 | (Misof et al. 2014) |
| 69 | Order | Coleoptera | Amino acids: 413459 | (Misof et al. 2014) |
| 70 | Suborder | Polyphaga | 1 nDNA, 2 mtDNA loci: 3784 bp | (Hunt et al. 2007) |
| 71 | Infraorder | Elateriformia | 1 nDNA, 2 mtDNA loci: 3784 bp | (Hunt et al. 2007) |
| 72 | Infraorder | Cucujiformia | 1 nDNA, 2 mtDNA loci: 3784 bp | (Hunt et al. 2007) |
| 73 | Family | Melyridae | 2 nDNA, 2 mtDNA loci: 3705 bp | (Bocakova et al. 2012) |
| 74 | Superfamily | Tenebrionoidea | 2 nDNA, 2 mtDNA loci: 2082 bp | (Gunter et al. 2014) |
| 75 | Family | Cleridae | 2 nDNA, 2 mtDNA loci: 2082 bp | (Gunter et al. 2013) |
| 76 | Family | Mordellidae | 2 nDNA, 2 mtDNA loci: 2082 bp | (Gunter et al. 2014) |
| 77 | Family | Chrysomelidae | 2 nDNA, 1 mtDNA loci: 3000 bp | (Gómez‐Zurita et al. 2008) |
| 78 | Family | Curculionidae | 2 nDNA loci: 1940 bp | (Marvaldi et al. 2009) |
| 79 | Family | Coccinellidae | 2 nDNA, 3 mtDNA loci: 3315 bp | (Magro et al. 2010) |

**References**

Abbot, P. & Withgott, J.H., 2004. Phylogenetic and Molecular Evidence for Allochronic Speciation in Gall-Forming Aphids (Pemphigus). *Evolution*, 58(3), pp.539–553. Available at: http://onlinelibrary.wiley.com/doi/10.1111/j.0014-3820.2004.tb01677.x/abstract [Accessed November 21, 2011].

Benjamin, S. et al., 2008. Family ties: molecular phylogeny of crab spiders (Araneae: Thomisidae). *Cladistics*, 24, pp.708–722. Available at: http://onlinelibrary.wiley.com/doi/10.1111/j.1096-0031.2008.00202.x/full [Accessed March 6, 2013].

Blackledge, T.A. et al., 2009. Reconstructing web evolution and spider diversification in the molecular era. *Proceedings of the National Academy of Sciences*, 106(13), pp.5229–5234. Available at: http://www.pnas.org/content/106/13/5229.short [Accessed June 10, 2014].

Bocakova, M., Constantin, R. & Bocak, L., 2012. Molecular phylogenetics of the melyrid lineage (Coleoptera: Cleroidea). *Cladistics*, 28, pp.117–129. Available at: http://onlinelibrary.wiley.com/doi/10.1111/j.1096-0031.2011.00368.x/full [Accessed August 5, 2014].

Bosselaers, J. & Jocque, R., 2002. Studies in Corinnidae : cladistic analysis of 38 corinnid and liocranid genera , and transfer of Phrurolithinae. *Zoologica Scripta*, 31(3), pp.241–270. Available at: http://onlinelibrary.wiley.com/doi/10.1046/j.1463-6409.2002.00080.x/full [Accessed June 10, 2014].

Cameron, S.L. et al., 2004. Mitochondrial genome data alone are not enough to unambiguously resolve the relationships of Entognatha, Insecta and Crustacea sensu lato (Arthropoda). *Cladistics*, 20(6), pp.534–557. Available at: http://doi.wiley.com/10.1111/j.1096-0031.2004.00040.x.

Cryan, J.R. & Urban, J.M., 2012. Higher-level phylogeny of the insect order Hemiptera: is Auchenorrhyncha really paraphyletic? *Systematic Entomology*, 37(1), pp.7–21. Available at: http://doi.wiley.com/10.1111/j.1365-3113.2011.00611.x [Accessed August 1, 2014].

von Dohlen, C.D. & Moran, N.A., 1995. Molecular Phylogeny of the Homoptera: A Paraphyletic Taxon. *Journal of Molecular Evolution*, 41(2), pp.211–223. Available at: http://www.springerlink.com/index/X818727416432690.pdf [Accessed January 10, 2011].

Von Dohlen, C.D., Rowe, C. a. & Heie, O.E., 2006. A test of morphological hypotheses for tribal and subtribal relationships of Aphidinae (Insecta: Hemiptera: Aphididae) using DNA sequences. *Molecular Phylogenetics and Evolution*, 38(2), pp.316–29. Available at: http://www.ncbi.nlm.nih.gov/pubmed/16368250 [Accessed July 9, 2011].

Gómez‐Zurita, J., Hunt, T. & Vogler, A., 2008. Multilocus ribosomal RNA phylogeny of the leaf beetles (Chrysomelidae). *Cladistics*, 24, pp.34–50. Available at: http://onlinelibrary.wiley.com/doi/10.1111/j.1096-0031.2007.00167.x/full [Accessed August 5, 2014].

Goropashnaya, A. V et al., 2012. Phylogenetic relationships of Palaearctic Formica species (Hymenoptera, Formicidae) based on mitochondrial cytochrome B sequences. *PloS one*, 7(7), p.e41697. Available at: http://www.pubmedcentral.nih.gov/articlerender.fcgi?artid=3402446&tool=pmcentrez&rendertype=abstract [Accessed July 11, 2014].

Grazia, J., Schuh, R.T. & Wheeler, W.C., 2008. Phylogenetic relationships of family groups in Pentatomoidea based on morphology and DNA sequences (Insecta: Heteroptera). *Cladistics*, 24(6), pp.932–976. Available at: http://doi.wiley.com/10.1111/j.1096-0031.2008.00224.x.

Gunter, N.L. et al., 2013. A molecular phylogeny of the checkered beetles and a description of Epiclininae a new subfamily (Coleoptera: Cleroidea: Cleridae). *Systematic Entomology*, 38(3), pp.626–636. Available at: http://doi.wiley.com/10.1111/syen.12019 [Accessed August 3, 2014].

Gunter, N.L. et al., 2014. Towards a phylogeny of the Tenebrionoidea (Coleoptera). *Molecular phylogenetics and evolution*, 79, pp.305–312. Available at: http://www.ncbi.nlm.nih.gov/pubmed/25053567 [Accessed August 3, 2014].

Hedtke, S.M., Patiny, S. & Danforth, B.N., 2013. The bee tree of life: a supermatrix approach to apoid phylogeny and biogeography. *BMC evolutionary biology*, 13, p.138. Available at: http://www.pubmedcentral.nih.gov/articlerender.fcgi?artid=3706286&tool=pmcentrez&rendertype=abstract [Accessed July 21, 2014].

Hunt, T. et al., 2007. A comprehensive phylogeny of beetles reveals the evolutionary origins of a superradiation. *Science (New York, N.Y.)*, 318(5858), pp.1913–1916. Available at: http://www.ncbi.nlm.nih.gov/pubmed/18096805 [Accessed July 12, 2014].

Karsholt, O. et al., 2013. A molecular analysis of the Gelechiidae (Lepidoptera, Gelechioidea) with an interpretative grouping of its taxa. *Systematic Entomology*, 38, pp.334–348. Available at: http://onlinelibrary.wiley.com/doi/10.1111/syen.12006/full [Accessed August 5, 2014].

Kutty, S.N. et al., 2008. The Muscoidea (Diptera: Calyptratae) are paraphyletic: Evidence from four mitochondrial and four nuclear genes. *Molecular phylogenetics and evolution*, 49(2), pp.639–52. Available at: http://www.ncbi.nlm.nih.gov/pubmed/18793735 [Accessed August 21, 2014].

Leppänen, S. a et al., 2012. Phylogenetics and evolution of host-plant use in leaf-mining sawflies (Hymenoptera: Tenthredinidae: Heterarthrinae). *Molecular phylogenetics and evolution*, 64(2), pp.331–41. Available at: http://www.ncbi.nlm.nih.gov/pubmed/22531610 [Accessed July 21, 2014].

Li, M. et al., 2012. Higher level phylogeny and the first divergence time estimation of Heteroptera (Insecta: Hemiptera) based on multiple genes. *PloS one*, 7(2), p.e32152. Available at: http://www.pubmedcentral.nih.gov/articlerender.fcgi?artid=3288068&tool=pmcentrez&rendertype=abstract [Accessed September 29, 2014].

Maddison, W.P. & Hedin, M.C., 2003. Jumping spider phylogeny (Araneae : Salticidae). *Invertebrate Systematics*, 17(4), pp.529–549. Available at: http://www.publish.csiro.au/?paper=IS02044.

Magro, A. et al., 2010. Phylogeny of ladybirds (Coleoptera: Coccinellidae): are the subfamilies monophyletic? *Molecular phylogenetics and evolution*, 54(3), pp.833–48. Available at: http://www.ncbi.nlm.nih.gov/pubmed/19903531 [Accessed July 21, 2014].

Marvaldi, A.E. et al., 2009. Structural alignment of 18S and 28S rDNA sequences provides insights into phylogeny of Phytophaga (Coleoptera: Curculionoidea and Chrysomeloidea). *Zoologica Scripta*, 38(1), pp.63–77. Available at: http://doi.wiley.com/10.1111/j.1463-6409.2008.00360.x [Accessed July 15, 2014].

Misof, B. et al., 2014. Phylogenomics resolves the timing and pattern of insect evolution. *Science*, 346(6210), pp.763–767. Available at: http://www.sciencemag.org/cgi/doi/10.1126/science.1257570 [Accessed November 6, 2014].

Munro, J.B. et al., 2011. A molecular phylogeny of the Chalcidoidea (Hymenoptera). *PloS one*, 6(11), p.e27023. Available at: http://www.pubmedcentral.nih.gov/articlerender.fcgi?artid=3207832&tool=pmcentrez&rendertype=abstract [Accessed July 21, 2014].

Ortiz-Rivas, B. & Martínez-Torres, D., 2010. Combination of molecular data support the existence of three main lineages in the phylogeny of aphids (Hemiptera: Aphididae) and the basal position of the subfamily Lachninae. *Molecular Phylogenetics and Evolution*, 55(1), pp.305–317. Available at: http://www.ncbi.nlm.nih.gov/pubmed/20004730 [Accessed August 6, 2011].

Ouellette, G.D., Fisher, B.L. & Girman, D.J., 2006. Molecular systematics of basal subfamilies of ants using 28S rRNA (Hymenoptera: Formicidae). *Molecular phylogenetics and evolution*, 40(2), pp.359–69. Available at: http://www.ncbi.nlm.nih.gov/pubmed/16630727 [Accessed July 21, 2014].

Quicke, D.L.J. et al., 2009. A thousand and one wasps: a 28S rDNA and morphological phylogeny of the Ichneumonidae (Insecta: Hymenoptera) with an investigation into alignment parameter space and elision. *Journal of Natural History*, 43(23–24), pp.1305–1421. Available at: http://www.tandfonline.com/doi/abs/10.1080/00222930902807783 [Accessed July 21, 2014].

Regier, J.C. et al., 2012. A molecular phylogeny for the leaf-roller moths (Lepidoptera: Tortricidae) and its implications for classification and life history evolution. *PloS one*, 7(4), p.e35574. Available at: http://www.pubmedcentral.nih.gov/articlerender.fcgi?artid=3334928&tool=pmcentrez&rendertype=abstract [Accessed July 11, 2014].

Scheffer, S.J., Winkler, I.S. & Wiegmann, B.M., 2007. Phylogenetic relationships within the leaf-mining flies (Diptera: Agromyzidae) inferred from sequence data from multiple genes. *Molecular phylogenetics and evolution*, 42(3), pp.756–75. Available at: http://www.ncbi.nlm.nih.gov/pubmed/17291785 [Accessed July 21, 2014].

Schnell e Schuehli, G., Barros De Carvalho, C.J. & Wiegmann, B.M., 2007. Molecular phylogenetics of the Muscidae (Diptera:Calyptratae): new ideas in a congruence context. *Invertebrate Systematics*, 21(3), p.263. Available at: http://www.publish.csiro.au/?paper=IS06026.

Schuh, R.T., Weirauch, C. & Wheeler, W.C., 2009. Phylogenetic relationships within the Cimicomorpha (Hemiptera: Heteroptera): a total-evidence analysis. *Systematic Entomology*, 34(1), pp.15–48. Available at: http://doi.wiley.com/10.1111/j.1365-3113.2008.00436.x.

Sharanowski, B.J., Dowling, A.P.G. & Sharkey, M.J., 2011. Molecular phylogenetics of Braconidae (Hymenoptera: Ichneumonoidea), based on multiple nuclear genes, and implications for classification. *Systematic Entomology*, 36, pp.549–572. Available at: http://onlinelibrary.wiley.com/doi/10.1111/j.1365-3113.2011.00580.x/full [Accessed August 14, 2014].

Sharkey, M.J. et al., 2012. Phylogenetic relationships among superfamilies of Hymenoptera. *Cladistics*, 28(1), pp.80–112.

Sihvonen, P. et al., 2011. Comprehensive molecular sampling yields a robust phylogeny for geometrid moths (Lepidoptera: Geometridae). *PLoS ONE*, 6(6), p.e20356. Available at: http://www.pubmedcentral.nih.gov/articlerender.fcgi?artid=3106010&tool=pmcentrez&rendertype=abstract [Accessed July 11, 2014].

Tian, Y. et al., 2008. Influence of data conflict and molecular phylogeny of major clades in Cimicomorphan true bugs (Insecta: Hemiptera: Heteroptera). *Molecular phylogenetics and evolution*, 47(2), pp.581–97. Available at: http://www.ncbi.nlm.nih.gov/pubmed/18396064.

Weirauch, C. & Munro, J.B., 2009. Molecular phylogeny of the assassin bugs (Hemiptera: Reduviidae), based on mitochondrial and nuclear ribosomal genes. *Molecular phylogenetics and evolution*, 53(1), pp.287–99. Available at: http://www.ncbi.nlm.nih.gov/pubmed/19531379 [Accessed August 3, 2014].

Wheeler, W.C. & Hayashi, C.Y., 1998. The Phylogeny of the Extant Chelicerate Orders. *Cladistics*, 14(2), pp.173–192. Available at: http://doi.wiley.com/10.1111/j.1096-0031.1998.tb00331.x.

Wieczorek, K. & Kajtoch, Ł., 2011. Relationships within Siphini (Hemiptera, Aphidoidea: Chaitophorinae) in light of molecular and morphological research. *Systematic Entomology*, 36(1), pp.164–174. Available at: http://doi.wiley.com/10.1111/j.1365-3113.2010.00550.x [Accessed July 21, 2014].

Wiegmann, B.M. et al., 2003. Time Flies, a New Molecular Time-Scale for Brachyceran Fly Evolution Without a Clock. *Systematic Biology*, 52(6), pp.745–756. Available at: http://journalsonline.tandf.co.uk/Index/10.1080/10635150390250965 [Accessed July 31, 2014].
