# Supplementary material for: Arthropod communities on hybrid and parental cottonwoods are phylogenetically structured by tree type: Implications for conservation of biodiversity in plant hybrid zones
Source: Ecol Evol. 2017 Jun 22;7(15):5909–21. doi: 10.1002/ece3.3146 (PMC5551273; doi:10.1002/ece3.3146)
Supplement: Supplementary file 2 [file ECE3-7-5909-s002.docx]

**Appendix 2.**

#NEXUS

[written Fri Nov 14 12:48:27 MST 2014 by Mesquite version 2.75 (build 566) at Karl-Jarviss-MacBook-Pro-2.local/192.168.1.139]

BEGIN TAXA;

TITLE Taxa;

DIMENSIONS NTAX=199;

TAXLABELS

Opi_1_1_1 Aca_Eriop_Aceria_parapopu Aca_Eryth_1_1 Ara_Dicty_Dictyna_1 Ara_Lycos_1_1 Ara_Oxyop_Oxyopes_1 Ara_Philo_Philodro_1 Ara_Theri_1_1 Ara_Theri_1_2 Ara_Clubi_Chiracan_1 Ara_Clubi_1_1 Ara_Clubi_1_2 Ara_Clubi_1_3 Ara_Arane_Araneus_1 Ara_Arane_Araniell_2 Ara_Arane_Metapeir_1 Ara_Arane_Metazygi_wittfeld Ara_Arane_Neoscona_1 Ara_Arane_1_1 Ara_Thomi_Misumeno_1 Ara_Thomi_Misumeno_2 Ara_Thomi_1_1 Ara_Thomi_1_2 Ara_Thomi_1_3 Ara_Thomi_1_4 Ara_Salti_Bredana_1 Ara_Salti_Corythal_1 Ara_Salti_Metacyrb_1 Ara_Salti_Metaphid_1 Ara_Salti_Metaphid_2 Ara_Salti_Paradamo_1 Ara_Salti_Phidippu_1 Ara_Salti_Salticus_1 Ara_Salti_Salticus_scenicus Ara_Salti_Talavera_1 Ara_Salti_1_4 Ara_Salti_1_5 Ara_Salti_1_6 Ara_Salti_1_7 Ara_Salti_1_8 Ara_Salti_1_9 Ara_Salti_1_10 Mic_1_1_1 Odo_Coena_1_1 Der_Forfi_1_1 Ort_Acrid_Melanopl_sanguini Ort_Gryll_Oecanthu_1 Ort_Tetti_1_1 Thy_Aeolo_1_1 Thy_Thrip_1_1 Thy_1_1_2 Hem_Diasp_Lepidosa_ulmi Hem_Aphid_Chaitoph_populico Hem_Aphid_Chaitoph_popullel Hem_Aphid_Thecabiu_populico Hem_Aphid_Thecabiu_populimo Hem_Aphid_Pemphigu_populigl Hem_Aphid_Pemphigu_populitr Hem_Aphid_Pemphigu_betae Hem_Aphid_Pemphigu_populica Hem_Cixii_1_1 Hem_Cerco_1_1 Hem_Cerco_1_2 Hem_Cicad_Gypona_1 Hem_Cicad_1_2 Hem_Cicad_1_3 Hem_Cicad_1_4 Hem_Cicad_1_5 Hem_Cicad_1_6 Hem_Cicad_1_7 Hem_Cicad_1_8 Hem_Cicad_1_10 Hem_Cicad_1_11 Hem_Cicad_1_12 Hem_Cicad_1_13 Hem_Cicad_1_14 Hem_Membr_1_1 Hem_Membr_1_2 Hem_Lygae_1_2 Hem_Rhopa_Leptocor_trivitta Hem_Scute_1_1 Hem_Penta_Apatetic_1 Hem_Penta_Brochyme_1 Hem_Penta_Euschist_1 Hem_Penta_Podisus_1 Hem_Penta_Thyanta_1 Hem_Reduv_Phymata_1 Hem_Reduv_Zelus_1 Hem_Reduv_1_1 Hem_Antho_Anthocor_antevole Hem_Nabid_1_1 Hem_Tingi_1_1 Hem_Mirid_Ceratoca_1 Hem_Mirid_1_1 Hem_Mirid_1_2 Hem_Mirid_1_3 Hem_Mirid_1_4 Hem_Mirid_1_5 Hem_Mirid_1_6 Hem_Mirid_1_7 Neu_Chrys_Argia_5 Neu_Raphi_1_1 Col_Anthi_1_1 Col_Bupre_1_1 Col_Canth_1_1 Col_Cicin_1_1 Col_Derme_1_1 Col_Lampy_1_1 Col_Scara_1_1 Col_Silph_1_1 Col_Teneb_1_1 Col_Cleri_1_1 Col_Cleri_1_2 Col_Morde_1_1 Col_Morde_1_2 Col_Melyr_1_1 Col_Melyr_1_2 Col_Melyr_1_3 Col_Melyr_1_4 Col_Chrys_Chrysome_confluen Col_Chrys_1_1 Col_Chrys_1_2 Col_Chrys_1_3 Col_Chrys_1_4 Col_Chrys_1_5 Col_Curcu_Microtyp_2 Col_Curcu_1_1 Col_Curcu_1_3 Col_Curcu_1_4 Col_Curcu_1_5 Col_Curcu_1_6 Col_Cocci_Adalia_bipuncta Col_Cocci_Coccinel_septum Col_Cocci_Cryptola_1 Col_Cocci_Harmonia_axyridis Col_Cocci_Hippodam_converge Col_Cocci_Scymnus_1 Col_Cocci_1_1 Col_Cocci_1_2 Hym_Chalc_1_1 Hym_Dryin_1_1 Hym_Encyr_1_1 Hym_Eulop_1_1 Hym_Eupel_1_2 Hym_Euryt_1_1 Hym_Ptero_1_1 Hym_Sphec_1_1 Hym_Vespi_1_1 Hym_Mymar_Stephano_1 Hym_Mymar_1_1 Hym_Tenth_Nematus_1 Hym_Tenth_Phylloco_1 Hym_Andre_1_1 Hym_Apida_1_1 Hym_Halic_1_1 Hym_Braco_1_2 Hym_Braco_1_3 Hym_Braco_1_4 Hym_Braco_1_5 Hym_Ichne_1_2 Hym_Ichne_1_3 Hym_Ichne_1_4 Hym_Ichne_1_5 Hym_Formi_Camponot_1 Hym_Formi_Tetramor_caespitu Hym_Formi_Formica_1 Hym_Formi_Formica_2 Hym_Formi_Formica_propinqu Lep_Graci_Phyllocn_1 Lep_Lyone_Paraleuc_albella Lep_Noctu_1_1 Lep_1_1_1 Lep_Gelec_Anacamps_niveopul Lep_Gelec_1_1 Lep_Geome_Alsophil_pometari Lep_Geome_1_1 Lep_Tortr_Gypsonom_haimbach Lep_Tortr_1_4 Lep_Tortr_1_5 Lep_Tortr_1_6 Dip_Antho_1_1 Dip_Bibio_1_1 Dip_Bomby_1_1 Dip_Calli_1_1 Dip_Cerat_1_1 Dip_Chiro_1_1 Dip_Chlor_1_1 Dip_Empid_1_1 Dip_Ottit_1_1 Dip_Phori_1_1 Dip_Sciar_1_1 Dip_Syrph_1_4 Dip_Tachy_1_1 Dip_Tephr_1_1 Dip_There_1_1 Dip_Agrom_Hexomyza_schineri Dip_Agrom_1_1 Dip_Musci_1_1 Dip_Musci_1_2

;

END;

BEGIN TREES;

Title Imported_trees;

LINK Taxa = Taxa;

TRANSLATE

1 Opi_1_1_1,

2 Aca_Eriop_Aceria_parapopu,

3 Aca_Eryth_1_1,

4 Ara_Dicty_Dictyna_1,

5 Ara_Lycos_1_1,

6 Ara_Oxyop_Oxyopes_1,

7 Ara_Philo_Philodro_1,

8 Ara_Theri_1_1,

9 Ara_Theri_1_2,

10 Ara_Clubi_Chiracan_1,

11 Ara_Clubi_1_1,

12 Ara_Clubi_1_2,

13 Ara_Clubi_1_3,

14 Ara_Arane_Araneus_1,

15 Ara_Arane_Araniell_2,

16 Ara_Arane_Metapeir_1,

17 Ara_Arane_Metazygi_wittfeld,

18 Ara_Arane_Neoscona_1,

19 Ara_Arane_1_1,

20 Ara_Thomi_Misumeno_1,

21 Ara_Thomi_Misumeno_2,

22 Ara_Thomi_1_1,

23 Ara_Thomi_1_2,

24 Ara_Thomi_1_3,

25 Ara_Thomi_1_4,

26 Ara_Salti_Bredana_1,

27 Ara_Salti_Corythal_1,

28 Ara_Salti_Metacyrb_1,

29 Ara_Salti_Metaphid_1,

30 Ara_Salti_Metaphid_2,

31 Ara_Salti_Paradamo_1,

32 Ara_Salti_Phidippu_1,

33 Ara_Salti_Salticus_1,

34 Ara_Salti_Salticus_scenicus,

35 Ara_Salti_Talavera_1,

36 Ara_Salti_1_4,

37 Ara_Salti_1_5,

38 Ara_Salti_1_6,

39 Ara_Salti_1_7,

40 Ara_Salti_1_8,

41 Ara_Salti_1_9,

42 Ara_Salti_1_10,

43 Mic_1_1_1,

44 Odo_Coena_1_1,

45 Der_Forfi_1_1,

46 Ort_Acrid_Melanopl_sanguini,

47 Ort_Gryll_Oecanthu_1,

48 Ort_Tetti_1_1,

49 Thy_Aeolo_1_1,

50 Thy_Thrip_1_1,

51 Thy_1_1_2,

52 Hem_Diasp_Lepidosa_ulmi,

53 Hem_Aphid_Chaitoph_populico,

54 Hem_Aphid_Chaitoph_popullel,

55 Hem_Aphid_Thecabiu_populico,

56 Hem_Aphid_Thecabiu_populimo,

57 Hem_Aphid_Pemphigu_populigl,

58 Hem_Aphid_Pemphigu_populitr,

59 Hem_Aphid_Pemphigu_betae,

60 Hem_Aphid_Pemphigu_populica,

61 Hem_Cixii_1_1,

62 Hem_Cerco_1_1,

63 Hem_Cerco_1_2,

64 Hem_Cicad_Gypona_1,

65 Hem_Cicad_1_2,

66 Hem_Cicad_1_3,

67 Hem_Cicad_1_4,

68 Hem_Cicad_1_5,

69 Hem_Cicad_1_6,

70 Hem_Cicad_1_7,

71 Hem_Cicad_1_8,

72 Hem_Cicad_1_10,

73 Hem_Cicad_1_11,

74 Hem_Cicad_1_12,

75 Hem_Cicad_1_13,

76 Hem_Cicad_1_14,

77 Hem_Membr_1_1,

78 Hem_Membr_1_2,

79 Hem_Lygae_1_2,

80 Hem_Rhopa_Leptocor_trivitta,

81 Hem_Scute_1_1,

82 Hem_Penta_Apatetic_1,

83 Hem_Penta_Brochyme_1,

84 Hem_Penta_Euschist_1,

85 Hem_Penta_Podisus_1,

86 Hem_Penta_Thyanta_1,

87 Hem_Reduv_Phymata_1,

88 Hem_Reduv_Zelus_1,

89 Hem_Reduv_1_1,

90 Hem_Antho_Anthocor_antevole,

91 Hem_Nabid_1_1,

92 Hem_Tingi_1_1,

93 Hem_Mirid_Ceratoca_1,

94 Hem_Mirid_1_1,

95 Hem_Mirid_1_2,

96 Hem_Mirid_1_3,

97 Hem_Mirid_1_4,

98 Hem_Mirid_1_5,

99 Hem_Mirid_1_6,

100 Hem_Mirid_1_7,

101 Neu_Chrys_Argia_5,

102 Neu_Raphi_1_1,

103 Col_Anthi_1_1,

104 Col_Bupre_1_1,

105 Col_Canth_1_1,

106 Col_Cicin_1_1,

107 Col_Derme_1_1,

108 Col_Lampy_1_1,

109 Col_Scara_1_1,

110 Col_Silph_1_1,

111 Col_Teneb_1_1,

112 Col_Cleri_1_1,

113 Col_Cleri_1_2,

114 Col_Morde_1_1,

115 Col_Morde_1_2,

116 Col_Melyr_1_1,

117 Col_Melyr_1_2,

118 Col_Melyr_1_3,

119 Col_Melyr_1_4,

120 Col_Chrys_Chrysome_confluen,

121 Col_Chrys_1_1,

122 Col_Chrys_1_2,

123 Col_Chrys_1_3,

124 Col_Chrys_1_4,

125 Col_Chrys_1_5,

126 Col_Curcu_Microtyp_2,

127 Col_Curcu_1_1,

128 Col_Curcu_1_3,

129 Col_Curcu_1_4,

130 Col_Curcu_1_5,

131 Col_Curcu_1_6,

132 Col_Cocci_Adalia_bipuncta,

133 Col_Cocci_Coccinel_septum,

134 Col_Cocci_Cryptola_1,

135 Col_Cocci_Harmonia_axyridis,

136 Col_Cocci_Hippodam_converge,

137 Col_Cocci_Scymnus_1,

138 Col_Cocci_1_1,

139 Col_Cocci_1_2,

140 Hym_Chalc_1_1,

141 Hym_Dryin_1_1,

142 Hym_Encyr_1_1,

143 Hym_Eulop_1_1,

144 Hym_Eupel_1_2,

145 Hym_Euryt_1_1,

146 Hym_Ptero_1_1,

147 Hym_Sphec_1_1,

148 Hym_Vespi_1_1,

149 Hym_Mymar_Stephano_1,

150 Hym_Mymar_1_1,

151 Hym_Tenth_Nematus_1,

152 Hym_Tenth_Phylloco_1,

153 Hym_Andre_1_1,

154 Hym_Apida_1_1,

155 Hym_Halic_1_1,

156 Hym_Braco_1_2,

157 Hym_Braco_1_3,

158 Hym_Braco_1_4,

159 Hym_Braco_1_5,

160 Hym_Ichne_1_2,

161 Hym_Ichne_1_3,

162 Hym_Ichne_1_4,

163 Hym_Ichne_1_5,

164 Hym_Formi_Camponot_1,

165 Hym_Formi_Tetramor_caespitu,

166 Hym_Formi_Formica_1,

167 Hym_Formi_Formica_2,

168 Hym_Formi_Formica_propinqu,

169 Lep_Graci_Phyllocn_1,

170 Lep_Lyone_Paraleuc_albella,

171 Lep_Noctu_1_1,

172 Lep_1_1_1,

173 Lep_Gelec_Anacamps_niveopul,

174 Lep_Gelec_1_1,

175 Lep_Geome_Alsophil_pometari,

176 Lep_Geome_1_1,

177 Lep_Tortr_Gypsonom_haimbach,

178 Lep_Tortr_1_4,

179 Lep_Tortr_1_5,

180 Lep_Tortr_1_6,

181 Dip_Antho_1_1,

182 Dip_Bibio_1_1,

183 Dip_Bomby_1_1,

184 Dip_Calli_1_1,

185 Dip_Cerat_1_1,

186 Dip_Chiro_1_1,

187 Dip_Chlor_1_1,

188 Dip_Empid_1_1,

189 Dip_Ottit_1_1,

190 Dip_Phori_1_1,

191 Dip_Sciar_1_1,

192 Dip_Syrph_1_4,

193 Dip_Tachy_1_1,

194 Dip_Tephr_1_1,

195 Dip_There_1_1,

196 Dip_Agrom_Hexomyza_schineri,

197 Dip_Agrom_1_1,

198 Dip_Musci_1_1,

199 Dip_Musci_1_2;

TREE Imported_tree_0 = ((1:4.0,(2:1.0,3:1.0)Acari:3.0,(4:3.0,5:3.0,6:3.0,7:3.0,(10:1.0,11:1.0,12:1.0,13:1.0):2.0,(20:1.0,21:1.0,22:1.0,23:1.0,24:1.0,25:1.0):2.0,((8:1.0,9:1.0):1.0,(14:1.0,15:1.0,16:1.0,17:1.0,18:1.0,19:1.0):1.0):1.0,(26:1.0,27:1.0,28:1.0,29:1.0,30:1.0,31:1.0,32:1.0,33:1.0,34:1.0,35:1.0,36:1.0,37:1.0,38:1.0,39:1.0,40:1.0,41:1.0,42:1.0):2.0)Araneae:1.0):10.5,(43:13.5,(44:12.5,((45:2.0,(46:1.0,47:1.0,48:1.0):1.0)Polyneoptera:9.5,(((49:1.0,50:1.0,51:1.0):7.0,((52:5.0,((53:1.0,54:1.0):3.0,(55:3.0,56:3.0,(57:2.0,58:2.0,(59:1.0,60:1.0):1.0):1.0):1.0):1.0)Sternorrhyncha:2.0,((61:3.0,((62:1.0,63:1.0):1.0,(64:1.0,65:1.0,66:1.0,67:1.0,68:1.0,69:1.0,70:1.0,71:1.0,72:1.0,73:1.0,74:1.0,75:1.0,76:1.0,77:1.0,78:1.0):1.0):1.0)Auchenorrhyncha:3.0,((79:3.0,80:3.0,(81:2.0,(82:1.0,83:1.0,84:1.0,85:1.0,86:1.0):1.0)Pentatomoidea:1.0)Pentatomorpha:2.0,((87:1.0,88:1.0,89:1.0):3.0,((90:1.0,91:1.0)Cimicoformes:2.0,(92:2.0,(93:1.0,94:1.0,95:1.0,96:1.0,97:1.0,98:1.0,99:1.0,100:1.0):1.0)Miriformes:1.0):1.0)Cimicomorpha:1.0)Heteroptera:1.0):1.0)Hemiptera:1.0)Hemimetabola:2.5,(((151:1.0,152:1.0):4.0,((147:3.0,148:3.0,(153:1.0,154:1.0,155:1.0):2.0,(164:2.0,165:2.0,(166:1.0,167:1.0,168:1.0):1.0):1.0)Aculeata:1.0,(((156:1.0,157:1.0,158:1.0,159:1.0):1.0,(160:1.0,161:1.0,162:1.0,163:1.0):1.0)Ichneumonoidea:1.0,((149:1.0,150:1.0):1.0,(140:1.0,141:1.0,142:1.0,143:1.0,144:1.0,145:1.0,146:1.0):1.0)Proctotrupomorpha:1.0):1.0)Apocrita:1.0)Hymenoptera:4.5,(((169:2.0,170:2.0,171:2.0,172:2.0,(173:1.0,174:1.0):1.0,(175:1.0,176:1.0):1.0,(177:1.0,178:1.0,179:1.0,180:1.0):1.0)Lepidoptera:4.0,(182:4.0,185:4.0,186:4.0,191:4.0,(183:3.0,184:3.0,187:3.0,188:3.0,189:3.0,190:3.0,192:3.0,193:3.0,194:3.0,195:3.0,(196:1.0,197:1.0):2.0,(181:2.0,(198:1.0,199:1.0):1.0):1.0)Brachycera:1.0)Diptera:2.0):2.5,((101:1.0,102:1.0):4.5,(106:5.0,(107:4.0,109:4.0,110:4.0,(104:1.0,105:1.0,108:1.0)Elateriformia:3.0,((116:1.0,117:1.0,118:1.0,119:1.0):2.0,(103:2.0,111:2.0,(112:1.0,113:1.0):1.0,(114:1.0,115:1.0):1.0)Tenebrionoidea:1.0,(120:1.0,121:1.0,122:1.0,123:1.0,124:1.0,125:1.0):2.0,(126:1.0,127:1.0,128:1.0,129:1.0,130:1.0,131:1.0):2.0,(132:1.0,133:1.0,134:1.0,135:1.0,136:1.0,137:1.0,138:1.0,139:1.0):2.0):1.0)Polyphaga:1.0)Coleoptera:0.5):3.0):1.0)Holometabola:1.0):1.0)Neoptera:1.0):1.0)Insecta:1.0):1.0[% ] [% ] [% setBetweenBits = selected ];

END;
