# Supplementary material for: Arthropod communities on hybrid and parental cottonwoods are phylogenetically structured by tree type: Implications for conservation of biodiversity in plant hybrid zones
Source: Ecol Evol. 2017 Jun 22;7(15):5909–21. doi: 10.1002/ece3.3146 (PMC5551273; doi:10.1002/ece3.3146)
Supplement: Supplementary file 3 [file ECE3-7-5909-s003.docx]

**Appendix 3.**

##########

# Import community arthropod data and phylogenies for community phylogenetic analyses

# Karl Jarvis June 13, 2014

##########

library(ape)

dir = "~/Projects/Phylomeet/Analysis_Misof_res/"

dataDir = paste0(dir, "arcot_data/")

treeDir = paste0(dir, "Trees/")

# number of randomized branch lengths

nrand = 9

##########

# Import phylogeny with ultrametricized branch lengths (from Mesquite)

##########

ultra = read.nexus(paste0(treeDir,"ultra.nex"))

ultra = ladderize(ultra, FALSE)

write.tree(ultra, paste0(treeDir,"ultra0.tre"))

ultras = list(ultra)

names(ultras) = "ultra"

edgeLength = length(ultra$edge.length)

# create more trees with randomly altered branch lengths

for(i in 1:nrand)

{

ultras[[i+1]] = ultra

ultras[[i+1]]$edge.length = ultra$edge.length + runif(edgeLength, min=-0.99, max=0.99)

names(ultras)[[i+1]] = paste0("urand", i)

write.tree(ultras[[i+1]], paste0(treeDir, "ultra", i, ".tre"))

}

##########

# Change branch lenths so each edge length is set equal

equal = ultra

equal$edge.length = rep(1, length=edgeLength)

equals = list(equal)

names(equals) = "equal"

write.tree(equal, paste0(treeDir, "equal0.tre"))

# Create trees with randomly altered branch lengths

for(i in 1:nrand)

{

equals[[i+1]] = equal

equals[[i+1]]$edge.length = runif(edgeLength, min=0.01, max=2)

names(equals)[[i+1]] = paste0("erand", i)

write.tree(equals[[i+1]], paste0(treeDir, "equal", i, ".tre"))

}

##########

# Combine all phylogenies into one list

phyloList = as.list(c(ultras, equals))

phyNames = names(phyloList)

plot(ultra, cex=0.5)

##################################

# Data preparation

# load picante package

library(picante)

dir = "~/Projects/Phylomeet/Analysis_Misof_res/"

# dir = "/scratch/kj375/Phylomeet/"

# Set directory for input data: community file and phylogeny file

dataDir = paste0(dir, "arcot_data/")

# Folder for phylogenies

treeDir = paste0(dir, "Trees/")

# Set folders for output

PDdir = paste0(dir, "Results_PD/")

MPDdir = paste0(dir, "Results_MPD/")

NRIdir = paste0(dir, "Results_NRI/")

comDistDir = paste0(dir, "Results_ComDist/")

# Set directory for figures

figDir <- paste0(dir, "Figures/")

##########

# Load, pool, and organize community data into data structures

##########

# load community data

crosstypes = c("fr","fo","na")

years = c(2000:2003)

types = rep(crosstypes, 4)

yrs = rep(years, each=3)

fullCom = read.table(paste(dataDir, 'arcot.txt', sep=''))

crosstype = factor(rep(rep(c('fr','fo','na','na'), each=10), times=4))

year = factor(rep(2000:2003, each=40))

comdf = cbind(year, crosstype, fullCom)

# pool occurrences by year and by crosstype

CtYrMaker = function(com)

{

fr2000 = apply(com[com$year==2000 & com$crosstype=='fr',3:ncol(com)],2,sum)

fo2000 = apply(com[com$year==2000 & com$crosstype=='fo',3:ncol(com)],2,sum)

na2000 = apply(com[com$year==2000 & com$crosstype=='na',3:ncol(com)],2,sum)

fr2001 = apply(com[com$year==2001 & com$crosstype=='fr',3:ncol(com)],2,sum)

fo2001 = apply(com[com$year==2001 & com$crosstype=='fo',3:ncol(com)],2,sum)

na2001 = apply(com[com$year==2001 & com$crosstype=='na',3:ncol(com)],2,sum)

fr2002 = apply(com[com$year==2002 & com$crosstype=='fr',3:ncol(com)],2,sum)

fo2002 = apply(com[com$year==2002 & com$crosstype=='fo',3:ncol(com)],2,sum)

na2002 = apply(com[com$year==2002 & com$crosstype=='na',3:ncol(com)],2,sum)

fr2003 = apply(com[com$year==2003 & com$crosstype=='fr',3:ncol(com)],2,sum)

fo2003 = apply(com[com$year==2003 & com$crosstype=='fo',3:ncol(com)],2,sum)

na2003 = apply(com[com$year==2003 & com$crosstype=='na',3:ncol(com)],2,sum)

out = rbind(fr2000,fo2000,na2000,fr2001,fo2001,na2001,fr2002,fo2002,na2002,fr2003,fo2003,na2003)

out

}

CtYr = CtYrMaker(com=comdf)

# pool occurrences by crosstype

CtMaker = function(com)

{

fr = apply(com[com$crosstype=='fr',3:ncol(com)],2,sum)

fo = apply(com[com$crosstype=='fo',3:ncol(com)],2,sum)

na = apply(com[com$crosstype=='na',3:ncol(com)],2,sum)

out = rbind(fr, fo, na)

out

}

Ct = CtMaker(com=comdf)

# list of communities to use in this analysis

comList = lapply(list(fullCom, CtYr, Ct), as.matrix)

names(comList) = c('indiv', 'pooled', 'crosstype')

CtYrNames <- c("fr2000","fo2000","na2000","fr2001","fo2001","na2001","fr2002","fo2002","na2002","fr2003","fo2003","na2003")

spaces <- c(0.2,0.2,0.2,1,0.2,0.2,1,0.2,0.2,1,0.2,0.2)

##########

# Load in phylogenies, prune, and create distance matrices for analysis

##########

# Read in phylogenies

phyleNames = list.files(treeDir, pattern='[.]tre')

phyNames = gsub('.tre','',phyleNames)

phyList = vector("list", length(phyNames))

names(phyList) = gsub('.tre','',phyleNames)

for(i in 1:length(phyleNames))

{

phyList[[i]] = read.tree(paste0(treeDir, phyleNames[i]))

}

# Prune phylogenies by community

phyPrune = vector('list',length=length(comList))

names(phyPrune) = names(comList)

for (i in 1:length(comList))

{

phyPrune[[i]] = vector('list', length=length(phyNames))

names(phyPrune[[i]]) = phyNames

for (j in 1:length(phyNames))

{

phyPrune[[i]][[j]] = prune.sample(comList[[i]], phyList[[j]])

}

}

# Create distance matrix for each topology

phyDist = vector('list', length(comList))

names(phyDist) = names(comList)

for (i in 1:length(comList))

{

phyDist[[i]] = vector('list', length(phyNames))

names(phyDist[[i]]) = phyNames

for (j in 1:length(phyNames))

{

phyDist[[i]][[j]] = cophenetic(phyPrune[[i]][[j]])

}

}

##########

# Faith's Phylogenetic Diversity (PD)

##########

PD = vector("list", length(comList))

names(PD) = names(comList)

for (i in 1:length(comList))

{

PD[[i]] = vector("list", length(phyNames))

for (j in 1:length(phyList))

{

PD[[i]][[j]] = pd(comList[[i]], phyList[[j]], include.root=TRUE)

write.csv(PD[[i]][[j]], paste0(PDdir, "PD_", names(comList)[i], "_", phyNames[j], ".csv"))

print(j)

}

}

##########

# Mean Phylogenetic Distance (MPD) and Net Relatedness Index (NRI)

##########

NRI_abund = NRI_pres = vector("list", length(comList))

for (i in 1:length(comList))

{

NRI_abund[[i]] = NRI_pres[[i]] = vector("list", length(phyNames))

for (j in 1:length(phyList))

{

NRI_abund[[i]][[j]] = ses.mpd(comList[[i]], phyDist[[i]][[j]], null.model="richness", abundance.weighted=T, runs = 999)

NRI_pres[[i]][[j]] = ses.mpd(comList[[i]], phyDist[[i]][[j]], null.model="richness", abundance.weighted=F, runs = 999)

write.csv(NRI_abund[[i]][[j]], paste0(NRIdir, "NRI_abund_", names(comList)[i],"_", phyNames[j], ".csv"))

write.csv(NRI_pres[[i]][[j]], paste0(NRIdir, "NRI_pres_", names(comList)[i],"_", phyNames[j], ".csv"))

print(j)

}

}

##########

# Phylogenetic Beta Diversity: Community Distance (ComDist)

##########

comDist_abund = comDist_pres = vector("list", length(comList))

names(comDist_abund) = names(comDist_pres) = names(comList)

for (i in 1:length(comList))

{

comDist_abund[[i]] = comDist_pres[[i]] = vector("list", length(phyNames))

names(comDist_abund[[i]]) = names(comDist_pres[[i]]) = phyNames

for (j in 1:length(phyList))

{

comDist_abund[[i]][[j]] = comdist(comList[[i]], phyDist[[i]][[j]], abundance.weighted=T)

comDist_pres[[i]][[j]] = comdist(comList[[i]], phyDist[[i]][[j]], abundance.weighted=F)

write.csv(as.matrix(comDist_abund[[i]][[j]]), paste0(comDistDir, "comDist_abund_", names(comList)[i], "_", phyNames[j], ".csv"))

write.csv(as.matrix(comDist_pres[[i]][[j]]), paste0(comDistDir, "comDist_pres_", names(comList)[i], "_", phyNames[j], ".csv"))

print(j)

}

}
